# Health Tract, No. 59

**Published:** 1867-04

**Authors:** 


### HEALTH TRACT, No. 59.

---

### CHECKING PERSPIRATION.

Edward Everett, the finished scholar, the accomplished diplomatist, the orator, the statesman, the patriot, became overheated in testifying in a court-room, on Monday morning, went to Fanueil Hall, which was cold, sat in a draft of air until his turn came to speak; "but my hands and feet were ice, my lungs on fire. In this condition I had to go and spend three hours in the court-room." He died in less than a week from this checking of the perspiration. It was enough to kill any man.

Professor Mitchel, the gallant soldier, and the most eloquent astronomical lecturer that has ever lived, while in a state of perspiration in yellow-fever, the certain sign of recovery, left his bed, went into another room, became chilled in a moment, and died the same night!

If while perspiring, or while something warmer than usual, from exercise or a heated room, there is a sudden exposure in stillness to a still, cold air, or to a raw, damp atmosphere, or to a draft, whether at an open window or door or street-corner, an inevitable result is a violent and instantaneous closing of the pores of the skin, by which waste and impure matters, which were making their way out of the system, are compelled to seek an exit through some other channel, and break through some weaker part, not the natural one, and harm to that part is the result. The idea is presented by saying that the cold has settled in that part. To illustrate:

A lady was about getting into a small boat to cross the Delaware; but wishing first to get an orange at a fruit-stand, she ran up the bank of the river, and on her return to the boat found herself much heated, for it was summer, but there was a little wind on the water, and the clothing soon felt cold to her; the next morning she had a severe cold, which settled on her lungs, and within the year she died of consumption.

A stout, strong man was working in a garden in May; feeling a little tired about noon, he sat down in the shade of the house and fell asleep; he waked up chilly; inflammation of the lungs followed, ending, after two years of great suffering, in consumption. On opening his chest, there was such an extensive decay, that the yellow matter was scooped out by the cupful.

A Boston ship-owner, while on the deck of one of his vessels, thought he would "lend a hand" in some emergency; and pulling off his coat, worked with a will, until he perspired freely, when he sat down to rest a while, enjoying the delicious breeze from the sea. On attempting to rise, he found himself unable, and was so stiff in his joints, that he had to be carried home and put to bed, which he did not leave until the end of two years, when he was barely able to hobble down to the wharf on crutches.

A lady, after being unusually busy all day, found herself heated and tired toward sundown of a summer's day. She concluded she would rest herself by taking a drive to town in an open vehicle. The ride made her uncomfortably cool, but she warmed herself up by an hour's shopping, when she turned homeward; it being late in the evening, she found herself more decidedly chilly than before. At midnight she had *pneumonia*, (inflammation of the lungs,) and in three months had the ordinary symptoms of confirmed consumption.

A lady of great energy of character lost her cook, and had to take her place for four days; the kitchen was warm, and there was a draft of air through it. When the work was done, warm and weary, she went to her chamber, and laid down on the bed to rest herself. This operation was repeated several times a day. On the fifth day she had an attack of lung fever; at the end of six months she was barely able to leave her chamber, only to find herself suffering with all the more prominent symptoms of confirmed consumption; such as quick pulse, night and morning cough, night-sweats, debility, short breath, and falling away.

A young lady rose from her bed on a November night, and leaned her arm on the cold window-sill to listen to a serenade. Next morning she had *pneumonia*, and suffered the horrors of asthma for the remainder of a long life.

Multitudes of women lose health and life every year, in one of two ways; by busying themselves in a warm kitchen until weary, and then throwing themselves on a bed or sofa, without covering, and perhaps in a room without fire; or by removing the outer clothing, and perhaps changing the dress for a more common one, as soon as they enter the house after a walk or a shopping. The rule should be invariable to go at once to a warm room and keep on all the clothing at least for five or ten minutes, until the forehead is perfectly dry. In all weathers, if you have to walk and ride on any occasion, do the riding first.

